# Effects of Rikkunshito treatment on renal fibrosis/inflammation and body weight reduction in a unilateral ureteral obstruction model in mice

**DOI:** 10.1038/s41598-020-58214-0

**Published:** 2020-02-05

**Authors:** Hiromichi Wakui, Takahiro Yamaji, Kengo Azushima, Kazushi Uneda, Kotaro Haruhara, Akiko Nakamura, Kohji Ohki, Sho Kinguchi, Ryu Kobayashi, Shingo Urate, Toru Suzuki, Daisuke Kamimura, Shintaro Minegishi, Tomoaki Ishigami, Tomohiko Kanaoka, Kohei Matsuo, Tomoyuki Miyazaki, Tetsuya Fujikawa, Akio Yamashita, Kouichi Tamura

**Affiliations:** 1 Department of Medical Science and Cardiorenal Medicine, Yokohama City University Graduate School of Medicine, Yokohama, Japan; 2 Cardiovascular and Metabolic Disorders Program, Duke-NUS Medical School, Singapore, Singapore; 3 Division of Nephrology and Hypertension, Department of Internal Medicine, The Jikei University School of Medicine, Tokyo, Japan; 4 Department of Physiology, Yokohama City University Graduate School of Medicine, Yokohama, Japan; 5 Center for Health Service Sciences, Yokohama National University, Yokohama, Japan; 6 Department of Molecular Biology, Yokohama City University Graduate School of Medicine, Yokohama, Japan

**Keywords:** Chronic kidney disease, Renal fibrosis

## Abstract

Chronic kidney disease (CKD) progresses to end-stage renal failure via renal tubulointerstitial fibrosis. Malnutrition, inflammation, and arteriosclerosis interact to exacerbate the poor prognosis of CKD, and their effective management is thus essential. The traditional Japanese medicine Rikkunshito (RKT) exerts appetite-stimulating effects via ghrelin, which attenuates inflammation and fibrosis. We evaluated the therapeutic effect of RKT in unilateral ureter obstruction (UUO)-induced renal fibrosis/inflammation and body weight loss in mice. UUO and sham-operated mice were fed a standard diet or diet containing 3.0% RKT. Renal fibrosis was investigated by histopathology and macrophage infiltration was determined by immunohistochemistry. Expression levels of genes associated with fibrosis, inflammation, ghrelin, and mitochondrial function were determined by quantitative reverse transcription-polymerase chain reaction and western blot analyses. RKT treatment partially prevented UUO-induced weight loss but failed to attenuate renal fibrosis and inflammation. Renal expression of sirtuin 1, a ghrelin-downstream signalling molecule, and gene expression of peroxisome proliferator-activated receptor-γ coactivator 1α and Bcl-2/adenovirus E1B interacting protein 3 were unaffected by RKT. These results indicate that RKT inhibits weight loss but does not improve renal fibrosis or inflammation in a rapidly progressive renal fibrosis mouse model. RKT may have a protective effect on weight loss associated with CKD.

## Introduction

The number of patients with chronic kidney disease (CKD) progressing to end-stage renal disease is increasing worldwide. Not only does CKD lead to end-stage renal disease, it is also associated with a high risk of cardiovascular disease and mortality. In patients with CKD, malnutrition, inflammation, and arteriosclerosis adversely affect each other and exacerbate their poor prognosis^1,2^. Therefore, the effective management of these pathological conditions is the key for the treatment of CKD.

Rikkunshito (RKT) is a traditional Japanese herbal medicine (Kampo medicine) that has historically been used to treat abnormalities of the digestive/nutrient absorption system. Ghrelin activation by RKT has been demonstrated as the mechanism responsible for the appetite-stimulating effects of RKT^3,4^. Ghrelin is a peptide hormone mainly secreted by the stomach and acts on the hypothalamus and digestive tract. It has important functions in the regulation of energy metabolism, such as the promotion of food intake, weight gain, and gastrointestinal function regulation^5–7^. Interestingly, ghrelin is also produced in the kidneys, and ghrelin receptors are expressed not only in the hypothalamus and gastrointestinal tract, but also in the cardiovascular tissues and kidneys^8,9^. This suggests that ghrelin has a variety of physiological activities beyond food intake promotion. In fact, it has been reported that in addition to possessing appetite-stimulating effects, ghrelin inhibits the nuclear factor-kappa B (NFκB) and transforming growth factor-β (TGFβ)/Smad3 cascades and so exerts both anti-inflammatory and anti-fibrotic effects^3,9,10^.

Therefore, RKT may have organ-protective effects by inhibiting inflammation/fibrosis via a ghrelin-activating action. Very recently, we have shown that RKT potentially exerts renal anti-inflammatory effects using angiotensin II (Ang II)-infused mice^11^. However, the Ang II-infused mouse model could not sufficiently elicit renal fibrosis to evaluate the effects of RKT on renal fibrosis. In the present study, we employed a mouse model of unilateral ureteral obstruction (UUO) to examine whether RKT treatment could suppress renal fibrosis.

## Materials and Methods

### Animals

This study was performed in accordance with the National Institutes of Health guidelines for the use of experimental animals. All animal experiments were reviewed and approved by the Animal Studies Committee of Yokohama City University. Mice were housed in a controlled environment with a 12-hour light–dark cycle at a temperature of 25 °C. Ten-week-old male C57BL/6 mice were divided into 4 groups as follows: (1) CTL-Sham group, sham-operated mice fed a control standard diet (0.6% NaCl and 3.4 kcal/g, CE-2; CLEA, Tokyo, Japan); (2) CTL-UUO group, unilateral ureteral obstruction (UUO)-operated mice fed a standard diet; (3) RKT-Sham group, sham-operated mice fed a diet containing RKT (CE-2 containing 3.0% RKT); and (4) RKT-UUO group, UUO-operated mice fed a diet containing RKT. RKT treatment groups were fed a diet containing RKT from 5 days before the operation.

The UUO procedure was performed using C57BL/6 mice, as described previously^12,13^. Briefly, with mice under anaesthesia, the left ureter was ligated at two locations. Mice receiving the procedure were killed under anaesthesia 7 days after UUO. Control animals received a sham operation, in which the ureters were manipulated but not ligated and were killed under the same conditions as those mice receiving UUO.

**Figure 1:**
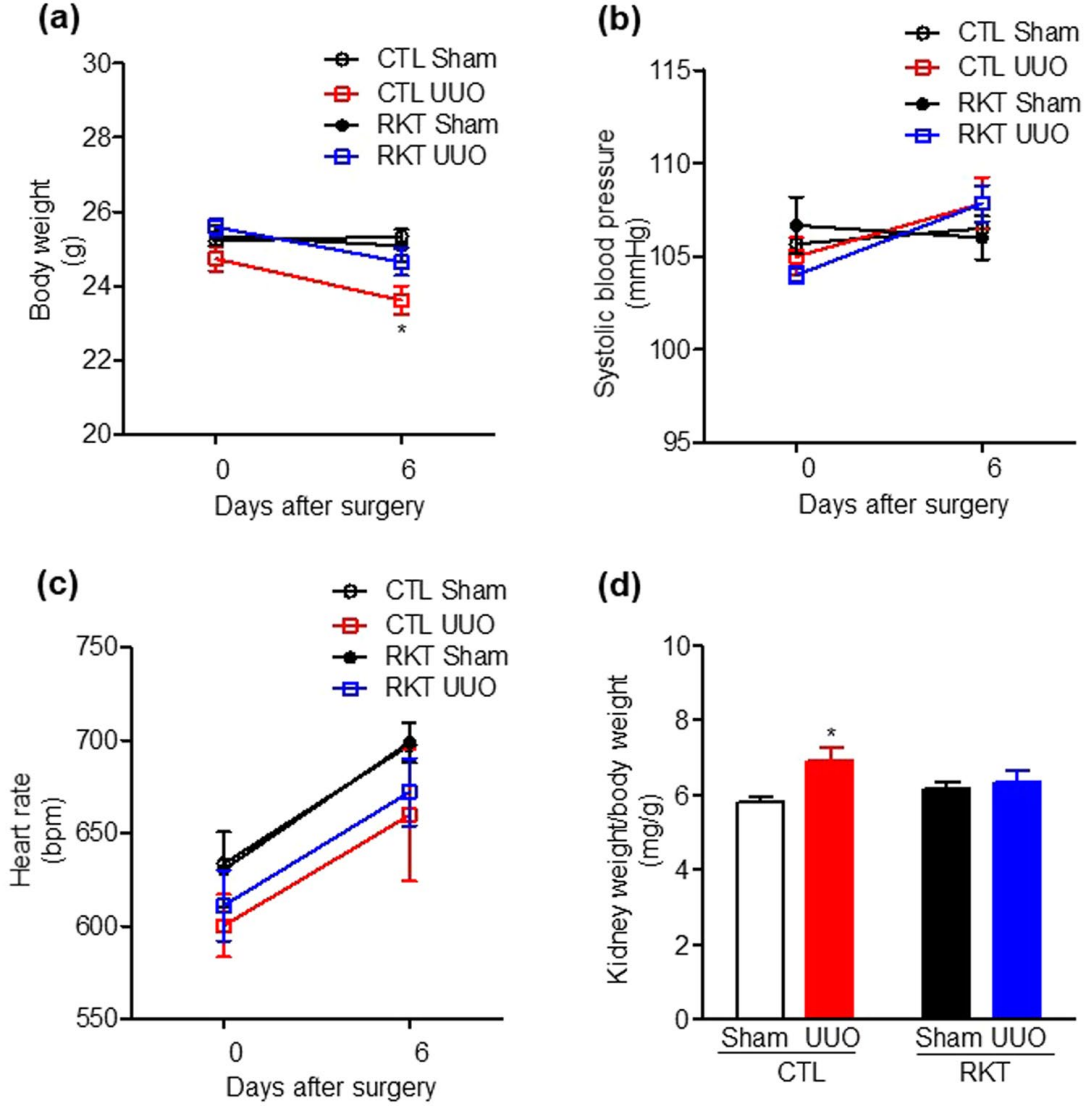
Effects of Rikkunshito treatment on body weight gain, blood pressure, heart rate, and the kidney weight/body weight ratio in sham- and UUO-operated mice. Effects of Rikkunshito treatment on body weight (**a**), systolic blood pressure (**b**), heart rate (**c**), and kidney weight/body weight ratio. (**d**) Values are expressed as the mean ± SE (n = 6 per group). Differences in body weight among the four groups over time were analysed by two-way repeated measures ANOVA with Bonferroni’s post-test. *P < 0.05 vs sham-operated group. CTL, control standard diet; RKT, diet containing Rikkunshito; UUO, unilateral ureteral obstruction.

**Figure 2:**
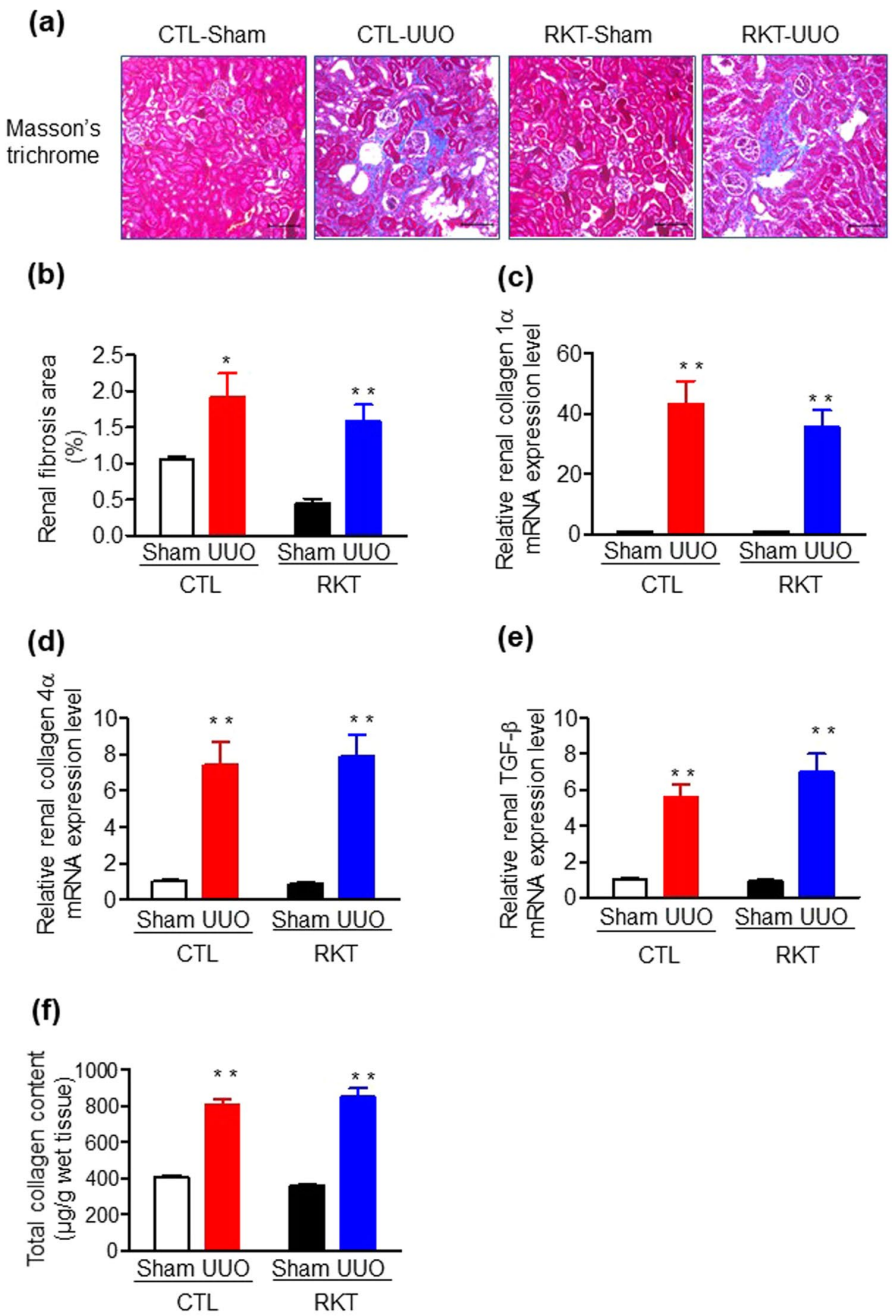
Effects of Rikkunshito treatment on renal fibrosis in sham- and UUO-operated mice. (**a**) Representative images of Masson’s trichrome staining in kidneys of sham- and UUO-operated mice fed a standard diet and a diet containing Rikkunshito. Original magnification: ×200. Scale bar = 100 μm. (**b**) Area of renal fibrosis in sham- and UUO-operated mice fed a standard diet and a diet containing Rikkunshito. (**c**–**e**) Quantitative analysis of fibrosis-related gene expression in kidney of sham- and UUO-operated mice fed a standard diet and a diet containing Rikkunshito. (**c**) Collagen 1α, (**d**) collagen 4α, and (**e**) TGF-β. (**f**) Total collagen assay for whole kidney in sham- and UUO-operated mice fed a standard diet and a diet containing Rikkunshito. Values are expressed as the mean ± SE (**a**–**e**, n = 6 in each group. **f**, n = 7–8 in each group). *P < 0.05, **P < 0.01 vs sham-operated group. Differences between sham and UUO mice within each diet and between CTL and RKT within each surgery were analysed by two-factor ANOVA with Bonferroni’s post-test. CTL, control standard diet; RKT, diet containing Rikkunshito; UUO, unilateral ureteral obstruction.

**Figure 3:**
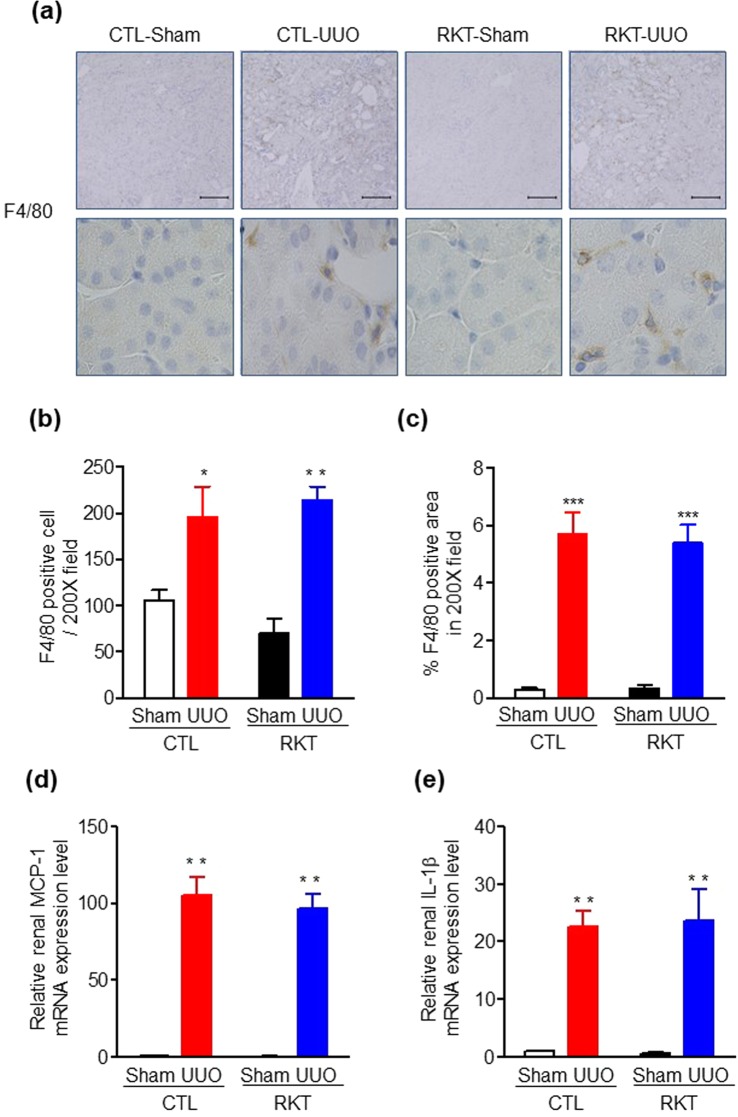
Effects of Rikkunshito treatment on renal inflammation in sham- and UUO-operated mice. (**a**) Representative images of F4/80 immunostaining in kidneys of sham- and UUO-operated mice fed a standard diet and a diet containing Rikkunshito. The positive areas for F4/80 are evident as brown dots in the sections. Original magnification, ×200 (upper panels), and ×400 (lower panels). Scale bar = 100 μm. (**b**) F4/80-positive cells and (**c**) % F4/80 positive area in kidneys of sham- and UUO-operated mice fed a standard diet and a diet containing Rikkunshito. Quantitative analysis of inflammation-related gene expression in kidney of sham- and UUO-operated mice fed a standard diet and a diet containing Rikkunshito. (**d**) Monocyte chemoattractant protein-1 (MCP-1) and (**e**) interleukin (IL)-1β. Values are expressed as mean ± SE (n = 6 per group). *P < 0.05, **P < 0.01 vs sham-operated group. Differences between sham and UUO mice within each diet and between CTL and RKT within each surgery were analysed by two-factor ANOVA with Bonferroni’s post-test. CTL, control standard diet; RKT, diet containing Rikkunshito; UUO, unilateral ureteral obstruction.

**Figure 4:**
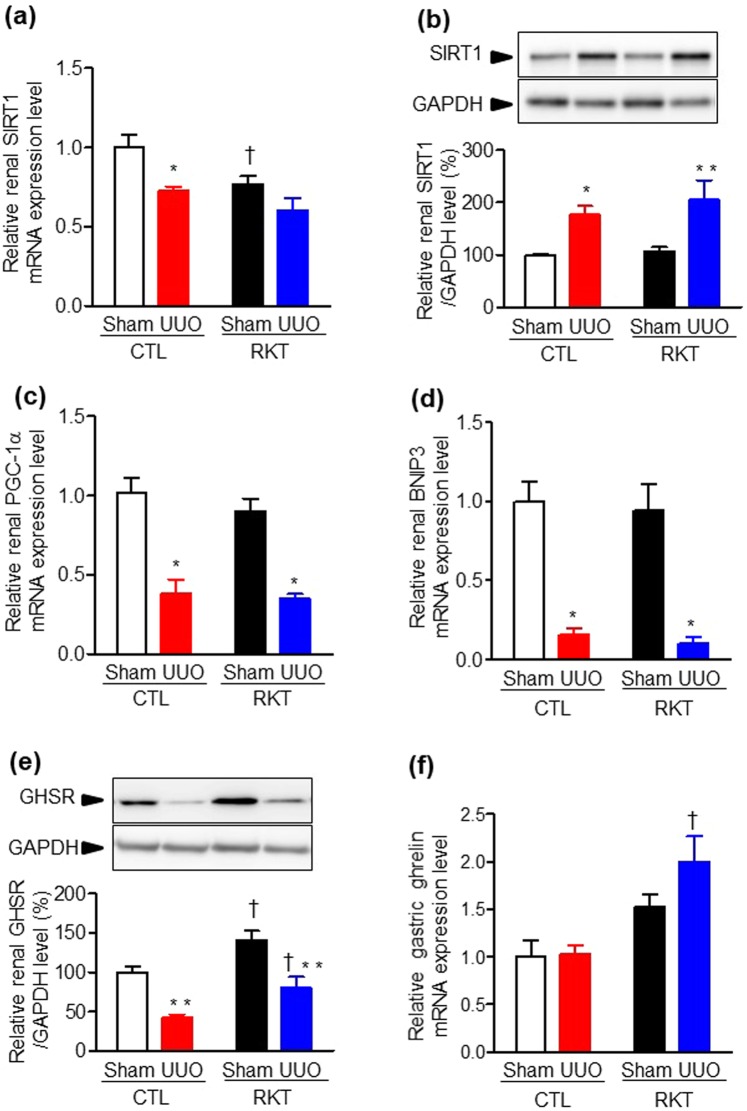
Effects of Rikkunshito treatment on renal level of SIRT1, mitochondrial function-related genes, GHSR, and gastric ghrelin levels in sham- and UUO-operated mice. Quantitative analysis of mRNA levels of (**a**) sirtuin 1 (SIRT1), (**c**) peroxisome proliferator-activated receptor-γ coactivator 1α (PGC-1α), (**d**) Bcl-2/adenovirus E1B interacting protein 3 (BNIP3) in kidneys, and (**f**) ghrelin in stomach of sham- and UUO-operated mice fed a standard diet and a diet containing Rikkunshito. Immunoblot analysis of (**b**) SIRT1 and (**e**) GHSR protein levels in kidneys of sham- and UUO-operated mice fed a standard diet and a diet containing Rikkunshito. Values are expressed as the mean ± SE (**a**–**e**, n = 6 in each group. **f**, n = 7–8 in each group). *P < 0.05, **P < 0.01 vs sham-operated group. ^†^*P* < 0.05 vs CTL group. Differences between sham and UUO mice within each diet and between CTL and RKT within each surgery were analysed by two-factor ANOVA with Bonferroni’s post-test. CTL, control standard diet; RKT, diet containing Rikkunshito; UUO, unilateral ureteral obstruction.

**Figure 5:**
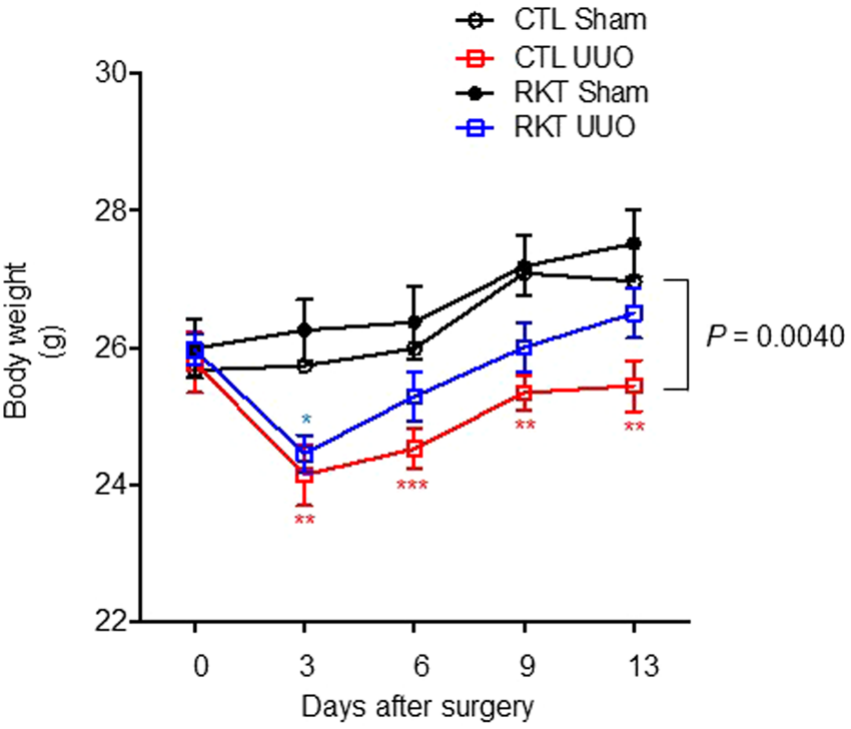
Effects of Rikkunshito treatment on body weight gain in sham- and UUO-operated mice. Values are expressed as mean ± SE (n = 6 per group). Differences in body weight among the four groups over time were analysed by two-way repeated measures ANOVA with Bonferroni’s post-test. *P < 0.05 vs sham-operated group fed a diet containing Rikkunshito. **P < 0.01, ***P < 0.001 vs sham-operated group fed a standard diet. CTL, control standard diet; RKT, diet containing Rikkunshito; UUO, unilateral ureteral obstruction.

The numbers of mice in each experiment are reported in the respective figure captions as follows: Fig. 1a–d, n = 6 per group; Fig. 2b–e, n = 6 per group; Fig. 2f, n = 7–8 per group; Fig. 3b–e, n = 6 per group; Fig. 4a–e, n = 6 per group; Fig. 4f, n = 7–8 per group; and Fig. 5, n = 6 per group.

### Treatment with rikkunshito

The Kampo medicine RKT is a dried powder prepared from the hot water extract of eight herbal drugs; Atractylodes rhizome (*Atractylodis lanceae rhizoma*), ginseng (*Ginseng radix*), Pinellia tuber (*Pinellia ternata*), Poria sclerotium (*Poria cocos*), jujube (*Zizyphi fructus*), citrus unshiu peel (*Aurantii nobilis pericarpium*), Glycyrrhiza (*Glycyrrhizae radix*), and ginger (*Zingiberis rhizoma*). The study diet was prepared by mixing powdered standard diet (CE-2; CLEA Japan) with RKT at a concentration of 3.0% RKT. RKT was obtained from Tsumura & Co. (Tokyo, Japan).

### Blood pressure measurement

Systolic blood pressure and heart rate were measured by the tail-cuff method (BP-Monitor MK-2000; Muromachi Kikai Co., Tokyo, Japan). The MK-2000 monitor enabled measurement of blood pressure without preheating the animals and using anaesthesia. This procedure avoided these stressful conditions, as described previously^14,15^. All measurements were performed between 10:00–14:00 hours. At least eight readings were taken for each measurement.

### Histological and immunohistochemical analyses

Kidneys were fixed with 4% paraformaldehyde and embedded in paraffin. Four µm thick sections were stained with haematoxylin and eosin, and Masson’s trichrome. Immunohistochemistry was performed as described previously^16–18^. Briefly, the paraffin-embedded sections were dewaxed and rehydrated. Antigen retrieval was performed by microwave heating in citrate buffer (16.2 ml 0.1 M sodium citrate solution, 3.8 ml 0.1 M citric acid solution, 80 ml distilled water). The sections were treated for 60 minutes with 10% normal goat serum in phosphate-buffered saline and blocked for endogenous biotin activity using an AVIDIN/BIOTIN Blocking kit (Vector Laboratories, Burlingame, CA, USA). The sections were then incubated with anti-F4/80 antibody (Abcam, Tokyo, Japan) diluted at 1:10. The sections were incubated for 60 minutes with biotinylated goat anti-rabbit IgG (Nichirei Corporation, Tokyo, Japan), blocked for endogenous peroxidase activity by incubation with 0.3% H_2_O_2_ for 20 minutes, treated for 30 minutes with streptavidin and biotinylated peroxidase (DAKO, Heidelberg, Germany), and then exposed to haematoxylin, dehydrated, and mounted. All images were acquired using a BZ-9000 microscope (Keyence, Osaka, Japan). The fibrotic area was analysed using Image J version 1.52e. The obtained image was red/blue/green divided and the blue was extracted. After binarization considering the overall balance, the tubular epithelium and the blue-stained part of the lumen were not fibrotic parts and were trimmed and excluded to quantify the fibrotic area. Perivascular fibrosis was included in the quantification. F4/80 positive cells stained the cytoplasm were counted visually and manually using an NDP.view 2 plus (Hamamatsu Photonics Co. Ltd., Hamamatsu, Japan) and the total positively staining area of each visual field was quantified as previously described^19^.

### Total collagen assay

Whole kidney samples were transferred into a screw-cap tube for hydrolysis and 1.0 ml 6 M hydrochloric acid was added per tube. The mixture was hydrolysed at 95 °C for 20 hours in a heat block, returned to room temperature, centrifuged at 13,000 × *g* for 10 minutes, and the supernatant was then used as a sample for measurement. The collagen concentration in each sample was measured using a Total Collagen Assay Kit (QuickZyme Biosciences, CK Leiden, Netherlands) according to manufacturer’s instructions.

### Real-time quantitative PCR analysis

Total RNA was extracted from the kidney with ISOGEN (Nippon Gene, Tokyo, Japan), and cDNA was synthesized using the SuperScript III First-Strand System (Invitrogen, Carlsbad, CA, USA). Real-time quantitative PCR (RT-qPCR) was performed with a Bio-Rad CFX96 Touch Real-Time PCR Detection System by incubating the reverse transcription product with TaqMan Universal PCR Master Mix and TaqMan probes (Applied Biosystems, Foster City, CA, USA), as described previously^20,21^. mRNA levels were normalized to 18S rRNA as a control. RT-qPCR was performed for 45 cycles, 18S was confirmed for expression around 15 cycles, and the target gene at 20–40 cycles.

### Immunoblot analysis

Immunoblot analysis was performed as described previously^11,22^. Briefly, total protein was extracted from kidney tissues with sodium dodecyl sulfate-containing sample buffer. The protein concentration of each sample was measured using a Detergent Compatible Protein Assay Kit (Bio-Rad, Tokyo, Japan). Equal amounts of protein extract were fractionated on a 5–20% polyacrylamide gel (ATTO Technology, Tokyo, Japan) and transferred to a polyvinylidene difluoride membrane using an iBlot Dry Blotting System (Invitrogen, MA, USA). The membranes were blocked for 1 hour at room temperature with phosphate-buffered saline containing 5% skim milk powder and then probed overnight at 4 °C with specific primary antibodies to sirtuin1 (SIRT1) (07–131; Merck Millipore, MA, USA), growth hormone secretagogue receptor (GHSR) (GHSR11-A; Alpha Diagnostic Intl. Inc., TX, USA), and glyceraldehyde 3-phosphate dehydrogenase (GAPDH) (ab8245; Abcam, Tokyo, Japan). SIRT1 antibody was diluted 1:1,000 with Signal Enhancer HIKARI for western blotting (Nacalai Tesque, Kyoto, Japan), GHSR antibody was diluted 1:1,000, and GAPDH antibody was diluted 1:5,000 with the same solution. Membranes were washed and further incubated with secondary antibodies for 15 minutes at room temperature. The sites of the antibody–antigen reaction were visualized by enhanced chemiluminescence substrate (GE Healthcare, Tokyo, Japan). Images were analysed quantitatively using a Fuji LAS-3000 image analyser (Fuji Film, Tokyo, Japan). A full blot summary is available in the Supplementary Information.

### Statistical analysis

Statistical analysis was performed using GraphPad Prism software (GraphPad Software, La Jolla, CA, USA). All quantitative data are expressed as the mean ± SEM. Differences in body weight among the four groups over time were analysed by two-way repeated measures ANOVA with Bonferroni’s post-test as previously described^23–26^. Differences between sham and UUO mice for each diet and between CTL and RKT within each surgery were tested by two-factor ANOVA with Bonferroni’s post-test. P-values of *P* < 0.05 were considered to indicate statistically significant differences.

## Results

### Effect of RKT on body weight, blood pressure, heart rate, and kidney weight in the UUO mouse model

Body weights before surgery were similar among the four groups (Fig. 1a). At 6 days after surgery, the body weight of the mice in the CTL-UUO group was significantly lower compared with the CTL-Sham group (two-way repeated measures ANOVA with Bonferroni’s post-test, *P* < 0.05) (Fig. 1a). However, there were no significant differences in body weights between the RKT-UUO and RKT-Sham groups and between the CTL-UUO and RKT-UUO groups. Systolic blood pressure and heart rate were identical among the four groups (Fig. 1b,c). At 6 days after surgery, kidney weight/body weight ratio in the CTL-UUO group was significantly increased compared with other groups (Fig. 1d). These results indicate that RKT treatment recovered the UUO-induced decrease in body weight.

### Effect of RKT on tubulointerstitial fibrosis in the UUO mouse model

We next examined tubulointerstitial fibrosis by histological analysis among the four groups of mice at 7 days after surgery. Tubulointerstitial fibrosis in the CTL-UUO and the RKT-UUO groups was significantly developed compared with the CTL-Sham and the RKT-Sham groups at 7 days post-surgery (Fig. 2a,b). However, there were no differences in tubulointerstitial fibrosis between the CTL-UUO and the RKT-UUO groups. In addition, we examined mRNA levels of renal fibrosis-related genes among the four groups at 7 days after surgery. The kidney expression of collagen 1α, collagen 4α, and TGF-β were all significantly increased in the CTL-UUO and the RKT-UUO groups compared with the CTL-Sham and the RKT-Sham groups (Fig. 2c–e). However, the upregulation of these genes was comparable between the CTL-UUO and the RKT-UUO groups. In addition, we examined collagen levels using a total collagen assay, as a standardized method for measuring fibrosis (Fig. 2f). Collagen levels were significantly higher in the CTL-UUO and RKT-UUO groups compared with the CTL-Sham and RKT-Sham groups at 7 days post-surgery (Fig. 2f). However, there were no significant differences in the amounts of collagen between the CTL-UUO and RKT-UUO groups. These results indicate RKT treatment does not suppress UUO-induced renal fibrosis.

### Effect of RKT on renal inflammation in the UUO mouse model

We next examined renal inflammation by immunohistochemical staining for F4/80 as a macrophage marker among the four groups at 7 days post-surgery. The macrophage infiltration in the kidney of CTL-UUO and RKT-UUO groups was significantly increased compared with the CTL-Sham and the RKT-Sham groups (Fig. 3a–c). There were no differences in macrophage infiltration between the CTL-UUO and the RKT-UUO groups. In addition, we examined mRNA levels of inflammation-related genes among the four groups at 7 days after surgery. The renal expressions of monocyte chemoattractant protein 1 (MCP-1, also known as CCL2) and interleukin (IL)-1β were significantly increased in the CTL-UUO and the RKT-UUO groups compared with the CTL-Sham and the RKT-Sham groups (Fig. 3d,e). However, upregulation of the expression of these genes was comparable between the CTL-UUO and the RKT-UUO groups. These results indicate RKT treatment does not suppress UUO-induced renal inflammation.

### Effect of RKT on renal expression of SIRT1, mitochondrial function-related genes and growth hormone secretagogue receptor in the UUO mouse model

As part of the downstream signalling pathway of the ghrelin receptor, we examined renal expressions of sirtuin 1 (SIRT1) among the four groups. Renal SIRT1 mRNA expression was significantly decreased in RKT-Sham compared with CTL-Sham mice (Fig. 4a), and in CTL-UUO compared with CTL-Sham mice (Fig. 4a). However, renal SIRT1 protein levels were comparable between the CTL-Sham and RKT-Sham groups (Fig. 4b). Renal SIRT1 protein expression was significantly increased in CTL-UUO and RKT-UUO mice compared with the CTL-Sham and RKT-Sham groups (Fig. 4b), but there was no significant difference between the CTL-UUO and RKT-UUO groups. These results indicated that SIRT1 mRNA and protein expression levels were inversely related, suggesting that UUO might affect the translation and/or stability of SIRT1 protein. We next examined the renal mRNA levels of mitochondrial function-related genes. The renal expression of peroxisome proliferator-activated receptor-γ coactivator 1α (PGC-1α) and Bcl-2/adenovirus E1B interacting protein 3 (BNIP3) were significantly decreased in the CTL-UUO and the RKT-UUO groups compared with the CTL-Sham and the RKT-Sham groups (Fig. 4c,d). However, downregulation of the expression of these genes was comparable between the CTL-UUO and the RKT-UUO groups. We also examined renal GHSR (ghrelin receptor) protein levels in the four groups (Fig. 4e). Renal expression of GHSR was significantly enhanced in RKT-Sham and RKT-UUO mice compared with the CTL-Sham and CTL-UUO groups. However, UUO significantly decreased renal GHSR expression in both the CTL and RKT groups by about 50%. We further examined gastric ghrelin mRNA levels in the four groups (Fig. 4f) and showed that RKT treatment significantly enhanced gastric expression levels of ghrelin (approximately 1.5–2.0 fold).

### Long-term effect of RKT on body weight in the UUO mouse model

To clarify the effect of RKT on body weight, we measured body weights more frequently for a longer time after surgery (Fig. 5). Body weights were significantly lower in CTL-UUO compared with CTL-Sham mice over time (two-way repeated measures ANOVA, *F* = 14.76, *P* = 0.0040) (Fig. 5). At 3 days after surgery, body weights were significantly lower in the CTL-UUO mice compared with the CTL-Sham mice (two-way repeated measures ANOVA with Bonferroni’s post-test, **P < 0.01 vs CTL-Sham group). In addition, body weights of the CTL-UUO mice remained significantly lower than those of the CTL-Sham mice until 13 days after surgery (two-way repeated measures ANOVA with Bonferroni’s post-test, **P < 0.01, ***P < 0.001 vs sham-operated group fed a standard diet). On the other hand, body weights tended to be lower in RKT-UUO compared with RKT-Sham mice over time, but the difference was not significant (two-way repeated measures ANOVA, *F* = 3.668, *P* = 0.0845). At post-operative day 3, body weights were significantly lower in the RKT-UUO mice compared with the RKT-Sham mice (two-way repeated measures ANOVA with Bonferroni’s post-test, *P < 0.05 vs RKT-Sham group). However, body weights of the RKT-UUO mice were quickly recovered afterwards. Body weights of the RKT-UUO mice were not statistically different from those of the sham-operated mice from day 6 to day 13 after surgery. In addition, body weights tended to be lower in CTL-UUO mice compared with RKT-UUO mice over time, although the difference was not significant (two-way repeated measures ANOVA, *F* = 2.712, *P* = 0.134). These results support the restorative effect of RKT on UUO-induced decreases in body weight.

## Discussion

The results of the present study showed that the administration of RKT potentially inhibited weight loss; however, it did not suppress renal fibrosis and inflammation in the mouse UUO model. UUO is a kidney disease model that causes renal fibrosis and inflammation. Renal tubulointerstitial fibrosis is known to be a final common pathway through which all renal diseases causing CKD eventually progress. Therefore, the UUO model is often used to evaluate the potential therapeutic effects of novel treatments on various kidney diseases. In the present study, UUO caused histologically significant renal fibrosis and greatly increased expression of fibrosis-related genes in mice. In addition, renal inflammation and the expression of inflammation-related genes were also significantly increased by UUO. These results suggest that kidney injury was appropriately induced by UUO in the present study. However, RKT treatment did not suppress histological renal fibrosis and inflammation, and even increased the expression of fibrosis/inflammation-related genes in response to UUO.

Traditional Kampo medicines are widely used in clinical practice in East Asia. Of these medicines, RKT has historically been used to treat abnormalities of the digestion/nutrient absorption system. It is also known to have an appetite promoting effect and facilitate weight gain under conditions of malnutrition. RKT promoted ghrelin secretion and ameliorated appetite reduction and weight loss in both human studies and cisplatin-induced anorexia animal models^27,28^. Furthermore, it was demonstrated that RKT improves ghrelin sensitivity in ghrelin-resistant pathologies, in which food intake does not increase despite the high circulating levels of ghrelin^29,30^. Ghrelin is mainly produced in gastric X/A-like cells. RKT reportedly elevated gene expression levels of gastric ghrelin and hypothalamic neuropeptide Y in rats^31^, and also increased mRNA expression levels of gastric ghrelin in mice^28,32^. In the present study, RKT-treated mice exhibited enhanced gastric levels of ghrelin mRNA, suggesting that RKT was effective in mice. Furthermore, an analysis of body weight changes after surgery revealed that UUO-induced weight loss was partially recovered by RKT treatment. Therefore, RKT treatment could exert a protective effect against weight loss in the UUO model.

A lower body mass index is associated with greater mortality in patients with CKD^33^. Low body weight is also closely associated with low lean mass (sarcopenia) in CKD. Sarcopenia is a chronic condition associated with aging and characterized by decreased muscle mass, strength, and function. Sarcopenia is prevalent in patients with CKD and is associated with increased risks of mortality and cardiovascular complications^34^. However, the pathogenesis of sarcopenia in CKD is complex and includes hormonal changes, metabolic acidosis, increased indoxyl sulfate, and inflammatory cytokines^35,36^. Although we did not examine skeletal muscle profiles and metabolism in the mice in the present study, RKT was shown to suppress weight loss in response to UUO. RKT treatment may therefore also improve sarcopenia associated with CKD. However, further studies are needed to determine the effect of RKT on skeletal muscle profiles and metabolism in the mouse UUO model.

In the present study, RKT did not suppress either renal fibrosis or inflammation caused by UUO. Recently, SIRT1 has been highlighted as a critical downstream pathway of ghrelin receptor signalling. Several *in vitro* studies have reported that ghrelin treatment elevated SIRT1 expression and/or activity in human umbilical vein endothelial cells, retinal microvascular endothelial cells, and cortical collecting duct cells^37–39^. SIRT1 is a key factor for the maintenance of mitochondrial biogenesis and functions via activation of PGC-1α by deacetylation^40,41^. Since the kidney is an organ with a high energy demand and is rich in mitochondria, mitochondrial dysfunction and subsequent reactive oxygen species generation in the kidney play an important role in the pathogenesis of kidney diseases. Fujitsuka *et al*. reported that RKT administration in a senescent mouse model activates SIRT1, inhibits inflammation and apoptosis associated with aging, and prolongs life span^37^. In addition, we have very recently reported that Ang II-induced decrease in renal SIRT1 expression was restored by RKT, concomitant with a suppression of renal inflammation in Ang II-infused mice^11^. In the present study, renal SIRT1 protein expression was antagonistically increased in response to UUO in mice. However, UUO provoked renal inflammation and fibrosis, concomitant with a decrease in renal expression of mitochondrial function-related genes. These results indicate that the UUO-induced renal inflammation and fibrosis were stronger than the renal protective effect of SIRT1. In addition, RKT treatment did not enhance the up-regulation of renal SIRT1 expression in response to UUO compared with the CTL group, and thus failed to improve renal fibrosis.

Why did RKT treatment not improve renal fibrosis and inflammation in the mouse model of UUO? One possibility is that UUO significantly reduced renal expression of GHSR (ghrelin receptor) in the present study. RKT was reported to increase GHSR expression in the hypothalamus in a cisplatin-induced anorexia model^42^. Intriguingly, RKT treatment in the present study significantly enhanced renal GHSR protein expression, but this RKT-induced increase was reduced in response to UUO. This downregulation of renal GHSR might thus have attenuated the renal protective action of RKT in the mouse UUO model. Furthermore, it should be noted that the pathogenesis of renal fibrosis in UUO is very rapid and causes severe fibrosis at local tissue sites. Traditionally, the beneficial effects of Kampo medicines in humans are mainly related to chronic diseases. Since the UUO model follows a rapid course from onset of hydronephrosis, the pathophysiology differs markedly from clinical human CKD, which progresses with a chronic course. The above-mentioned report of Fujitsuka *et al*. demonstrating anti-inflammatory effects and prolonged longevity via SIRT1 activation by RKT is a result of chronic administration of RKT over at least several months^37^. Therefore, to more appropriately examine the protective effects of RKT on renal fibrosis *in vivo*, it may be necessary to investigate another CKD model, such as aristolochic acid nephropathy or an adenine-overload nephropathy, which causes renal fibrosis along with a nutritional disorder over a more chronic course^43–45^.

The present study had some limitations. An assessment of renal fibrosis in the UUO model was performed only at one time point (day 7). Food consumption after UUO or sham surgery could not be accurately measured, and we were unable to evaluate whether ghrelin secretion was promoted by RKT administration.

In conclusion, RKT treatment potentially inhibited weight loss; however, it did not improve renal fibrosis and inflammation in mice with UUO, a rapidly progressive renal fibrosis model.

## Supplementary information

Full gel Western blot.

## Data Availability

The data used to support the findings of this study are included within the article.
