# Supplementary material for: Effects of Rikkunshito treatment on renal fibrosis/inflammation and body weight reduction in a unilateral ureteral obstruction model in mice
Source: Sci Rep. 2020 Feb 5;10:1782. doi: 10.1038/s41598-020-58214-0 (PMC7002622; doi:10.1038/s41598-020-58214-0)

**Supplementary information**

**Effects of Rikkunshito treatment on renal fibrosis/inflammation and body weight reduction in a unilateral ureteral obstruction model in mice**

Hiromichi Wakui^1*^, Takahiro Yamaji^1*^, Kengo Azushima^1,2^, Kazushi Uneda^1^, Kotaro Haruhara^1,3^, Akiko Nakamura^1^, Kohji Ohki^1^, Sho Kinguchi^1^, Ryu Kobayashi^1^, Shingo Urate^1^, Toru Suzuki^1^, Daisuke Kamimura^1^, Shintaro Minegishi^1^, Tomoaki Ishigami^1^, Tomohiko Kanaoka^1^, Kohei Matsuo^1^, Tomoyuki Miyazaki^4^, Tetsuya Fujikawa^5^, Akio Yamashita^6^ and Kouichi Tamura^1^

* H. Wakui and T. Yamaji contributed equally to this work.

^1^ Department of Medical Science and Cardiorenal Medicine, Yokohama City University Graduate School of Medicine, Yokohama, Japan; ^2^ Cardiovascular and Metabolic Disorders Program, Duke-NUS Medical School, Singapore; ^3^ Division of Nephrology and Hypertension, Department of Internal Medicine, The Jikei University School of Medicine, Tokyo, Japan; ^4^ Department of Physiology, Yokohama City University Graduate School of Medicine, Yokohama, Japan; ^5^ Center for Health Service Sciences, Yokohama National University, Yokohama Japan; ^6^ Department of Molecular Biology, Yokohama City University Graduate School of Medicine, Yokohama, Japan.

Correspondence to:

Hiromichi Wakui, M.D., Ph.D., or Kengo Azushima, M.D., Ph.D.

Department of Medical Science and Cardiorenal Medicine

Yokohama City University Graduate School of Medicine

3-9 Fukuura, Kanazawa-ku, Yokohama 236-0004, Japan.

Tel: 81-45-787-2635; Fax: 81-45-701-3738

E-mail: [hiro1234@yokohama-cu.ac.jp](mailto:hiro1234@yokohama-cu.ac.jp) or [azushima@yokohama-cu.ac.jp](mailto:azushima@yokohama-cu.ac.jp)


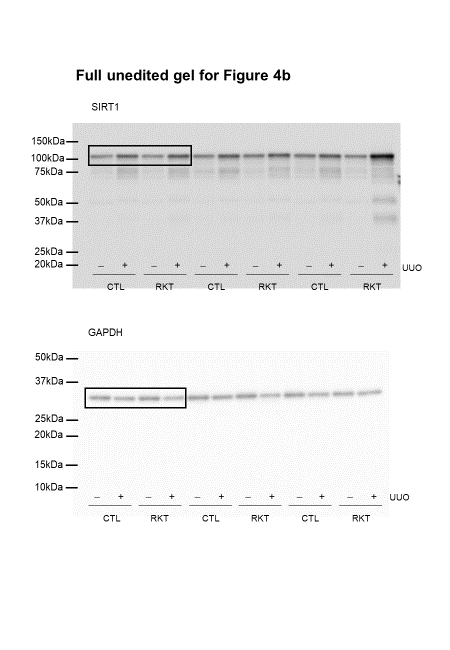


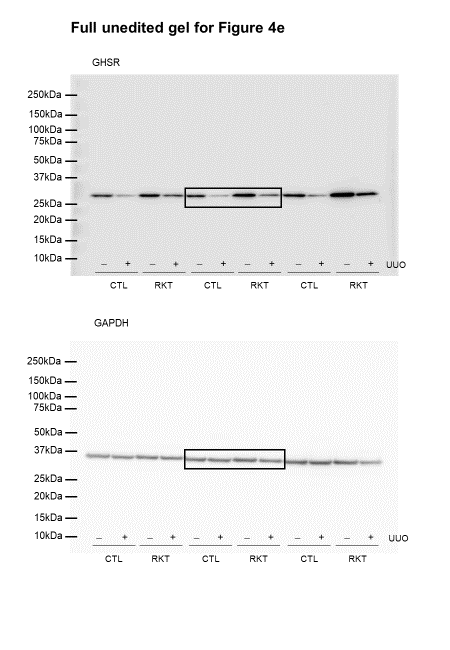

Supplement: Supplementary file 1 — Full gel Western blot. [file 41598_2020_58214_MOESM1_ESM.doc]
